# The Impact of Alcohol, Nicotine, and Opioid Use History on 90-Day and 1-Year Postoperative Complications Following Elective Laminectomy: A Nationwide Propensity-Matched Study

**DOI:** 10.1177/21925682251371593

**Published:** 2025-09-01

**Authors:** Zuhair Zaidi, Asim Mohamed, Billal Homayoun, Muaz Wahid, Salah Aoun, Michael Van Hal

**Affiliations:** 1 12334 UT Southwestern Medical School, Dallas, TX, USA; 2 Department of Neurological Surgery, 12334 UT Southwestern Medical Center, Dallas, TX, USA; 3 Department of Orthopaedic Surgery, 12334 UT Southwestern Medical Center, Dallas, TX, USA

**Keywords:** laminectomy, substance abuse, complications, alcohol, nicotine, opioids, elective surgery

## Abstract

**Study Design:**

Retrospective cohort study.

**Objectives:**

To assess the effect of alcohol, nicotine, and opioid use history on 90 day and 1 year postoperative complications after elective laminectomy.

**Methods:**

Using the TriNetX Database (2004-2025), adult patients undergoing elective laminectomy were divided into cohorts with a history of alcohol, nicotine, or opioid abuse/dependence, matched 1:1 with controls (age, sex, race, body mass index, comorbidities). Exclusions included malignancy, preceding trauma, and prior complications. Outcomes included the development of acute kidney failure, sepsis, emergency department visits, deep vein thrombosis, myocardial infarction, pulmonary embolism, pneumonia, stroke, and mortality at 90 days and 1 year.

**Results:**

Matched cohorts comprised 4053 alcohol, 22 469 nicotine, and 2724 opioid patients, each with controls. Alcohol abuse was significantly associated with increased odds of death, DVT, MI, pneumonia, sepsis, and stroke following elective laminectomy at 90 days, and with acute renal failure, PE, pneumonia, stroke, sepsis, DVT, and death at 1 year (*P* < 0.05). Nicotine dependence significantly elevated the risk of death, MI, pneumonia, and sepsis at 90 days, and at 1 year, remained associated with increased odds of acute renal failure, stroke, ED visits, MI, pneumonia, sepsis, and death (*P* < 0.05). Opioid abuse was significantly linked to higher risks of DVT, MI, pneumonia, sepsis, stroke, and death at 90 days, with persistent 1-year risks for acute renal failure, MI, pneumonia, sepsis, and death (*P* < 0.05).

**Conclusions:**

Alcohol, nicotine, and opioid use significantly heighten postoperative complications after laminectomy, underscoring the need for preoperative risk evaluation and tailored interventions.

## Introduction

Laminectomy, a key spinal decompression procedure, relieves spinal stenosis symptoms in middle-aged and elderly patients when nonoperative treatments fail. By excising vertebral components that have hypertrophied in response to degenerative changes in the spine, it promotes neural decompression, pain relief, and functional recovery. In the U.S., spinal stenosis affects many adults, with Medicare data showing 135.5-137.5 surgeries per 100 000 beneficiaries annually—a rate likely to grow with an aging population.^1^

To improve outcomes and reduce morbidity, identifying risk factors for postoperative complications is critical. Substance use disorders (SUDs)—alcohol, nicotine, and opioids—are major U.S. public health issues, affecting 14.5 million, 26.6 million, and 2.5 million adults, respectively.^2,3^ Research links SUDs to elevated perioperative risks in spinal surgery. Alcohol abuse correlates with a 10.15% multisystem complication rate in spinal fusion (33.73% with withdrawal) vs 6.14% in non-users, alongside infections, delirium, and mortality.^4,5^ Nicotine dependence increases sepsis, opioid use, ED visits, and revision surgeries in cervical fusion, with a 2.47 relative risk of revision after lumbar discectomy.^6^ Opioid use prolongs hospital stays, raises costs, and predicts prolonged postoperative dependence in lumbar fusion.^7^

Despite these findings, comprehensive data on SUDs’ impact on laminectomy outcomes are scarce, representing a key knowledge gap. This study uses a large U.S. multicenter database to analyze 90 day and 1 year postoperative effects of alcohol, nicotine, and opioid use in elective laminectomy patients, offering novel insights from an extensive national cohort.

## Methods

### Data Collection

This retrospective study utilized the TriNetX Health Research Network, aggregating deidentified electronic health record (EHR) data from over 124 million patients across 70 + healthcare organizations (60 in the U.S.) from 2004 to January 2, 2025.^8^ Compliant with the Health Insurance Portability and Accountability Act (HIPAA) and ISO 27001:2013, TriNetX provided diagnoses, procedures, medications, labs, and genomic data without requiring Institutional Review Board (IRB) approval due to its deidentified nature.

### Cohort Curation

Patients were identified using their respective ICD-10 codes for alcohol (F10.1, F10.2), nicotine (F17), or opioid (F11.1, F11.2) abuse/dependence. The index surgical procedures were limited to laminectomy, facetectomy, and foraminotomy, identified by the following CPT codes: 63 047 (lumbar), 63 046 (thoracic), 63 045 (cervical), 63 048 (additional segments), and 63 030 (lumbar laminotomy). Adults (≥18 years) with pre-procedure substance use history were included; those with other substance use were excluded to isolate effects. Exclusions comprised malignant neoplasms of the spinal cord or cauda equina (C72.0, C72.1), concurrent spinal fusion (CPT 1004098), recent trauma (within 1 month; M51.24–M51.27, S39.02, S39.82), or prior study complications.

The primary outcomes analyzed were acute kidney failure (N17), sepsis (A41.9), ED visits (CPT 1013711), DVT (I82.40), MI (I21), PE (I26), pneumonia (J13-J18), and cerebral infarction (stroke; I63) within 90 days and 1 year following laminectomy. Pneumonia outcomes included specific ICD-10-CM codes for bacterial, infectious, or unspecified causes (J13-J18). Patients with a history of these complications prior to the procedure were excluded to ensure that only new, postoperative occurrences were analyzed.

Baseline demographics (age, sex, race) and comorbidities (Essential Hypertension, Hyperlipidemia, Overweight and Obesity, Type 1 and 2 Diabetes Mellitus, Chronic Ischemic Heart Disease, Hypothyroidism, Chronic Kidney Disease, Polycystic Ovarian Syndrome) were recorded. Propensity score matching (1:1, nearest-neighbor greedy algorithm) adjusted for age, sex, race, BMI, hemoglobin A1c (HbA1c), and comorbidities.

### Statistical Analysis

TriNetX performed 1:1 propensity score matching; Prism 10 assessed outcomes. ORs with 95% CIs were calculated for complications. Chi-square and t-tests analyzed categorical and continuous variables, respectively.

### Cohort Selection

**Figure 1.**
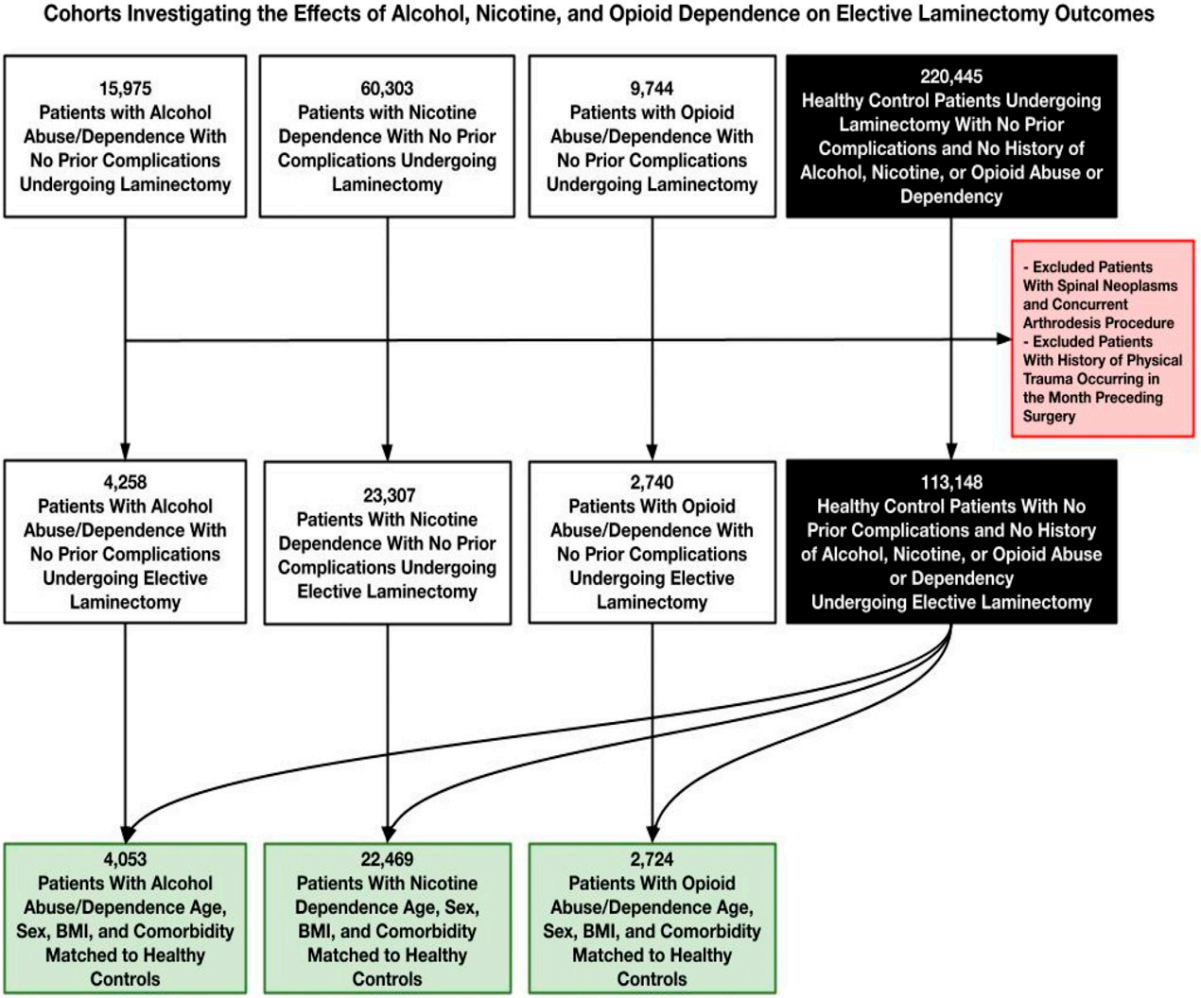
Flowchart Outlining Final Cohort Selection Process

Cohort selection began with a control group of 220 445 healthy patients undergoing laminectomy with no prior complications and no history of alcohol, nicotine, or opioid abuse or dependence. After applying exclusion criteria—such as spinal neoplasms, concurrent arthrodesis procedures, or physical trauma within the month preceding surgery—the control group was refined to 113 148 patients. Similarly, the substance use groups were refined using the same exclusion criteria, reducing the initial cohorts of 15 975 alcohol abuse patients, 60 303 nicotine dependence patients, and 9744 opioid abuse patients to smaller cohorts of 4 258, 23 307, and 2740 patients, respectively. These refined cohorts were then further matched by age, sex, BMI, and comorbidities, yielding final study groups of 4053 alcohol patients, 22 469 nicotine patients, and 2724 opioid patients, along with an equal number of matched healthy controls for each group. Figure 1 illustrates graphically the process of cohort selection.

## Results

### Baseline Patient Characteristics

**Figure 2.**
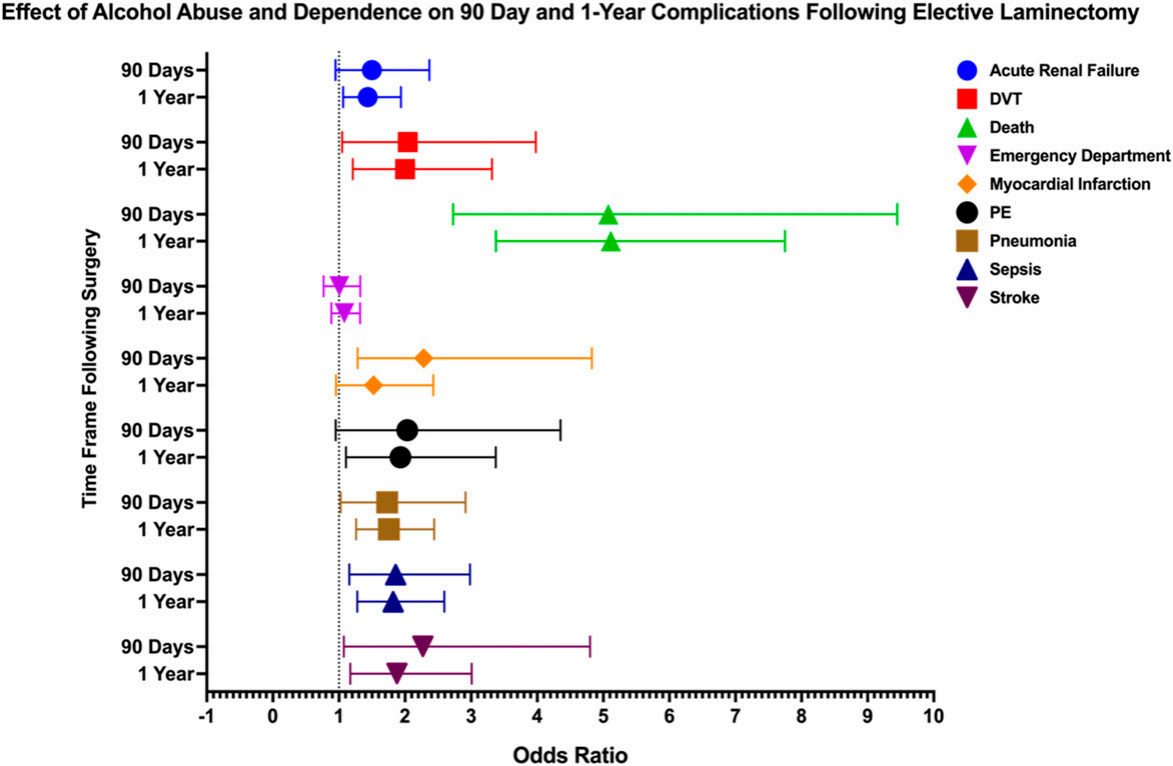
Forest Plot Depicting Impact of Alcohol Abuse and Dependence on 90 Day and 1 Year Postoperative Complications Following Elective Laminectomy

Table 1 summarizes the baseline characteristics of patients with and without SUDs who underwent elective laminectomy, after propensity score matching (PSM). After propensity score matching between the substance use cohorts and non-substance use healthy controls, the demographic characteristics were well-balanced, with no significant differences in age, sex, or BMI. The alcohol cohort had an average age of 58.1 ± 14.5 years compared to 58.1 ± 14.8 years in controls (*P* = 0.93), while the nicotine cohort averaged 54.9 ± 14.4 years vs 54.7 ± 14.6 years in controls (*P* = 0.45). Similarly, the opioid cohort showed comparable age distributions, with cases averaging 55.7 ± 14.6 years and controls 55.4 ± 14.8 years (*P* = 0.74). BMI values were also similar across cohorts, with the alcohol cohort at 29.4 ± 6.18 vs 31.1 ± 6.39 (*P* = 0.444), the nicotine cohort at 30.1 ± 6.6 vs 30.8 ± 6.5 (*P* = 0.32), and the opioid cohort at 30.4 ± 6.93 vs 31.7 ± 6.62 (*P* = 0.35). The sex distribution was also well-matched, with males comprising 70.4% of the alcohol cohort compared to 70.1% of controls (*P* = 0.7) and females comprising 25.5% vs 25.9% (*P* = 0.63). In the nicotine cohort, males accounted for 57.8% vs 58.1% (*P* = 0.52), and females 36.6% vs 36.4% (*P* = 0.62). Similarly, the opioid cohort had 52.1% males and 42.4% females compared to 53.1% and 41.6% in controls (*P* = 0.53 and *P* = 0.62, respectively) (Figure 2).

**Table 1.** Baseline Characteristics of Patients With or Without (Control) a Prior History of SUDs Who Received an Elective Laminectomy After PSM

After propensity score matching									
Baseline patient characteristics	Alcohol cohort (*n* = 4053)	Controls (*n* = 4053)	*P*-Value	Nicotine cohort (*n* = 22 469)	Controls (*n* = 22 469)	*P*-Value	Opioid cohort (*n* = 2724)	Controls (*n* = 2724)	*P*-Value
Age at index	58.1 ± 14.5	58.1 ± 14.8	0.93	54.9 ± 14.4	54.7 ± 14.6	0.45	55.7 ± 14.6	55.4±14.8	0.74
Sex									
Male	70.4%	70.1%	0.7	57.8%	58.1%	0.52	52.1%	53.1%	0.53
Female	25.5%	25.9%	0.63	36.6%	36.4%	0.62	42.4%	41.6%	0.62
Unknown gender	4.17%	4.1%	0.87	5.57%	5.55%	0.73	5.47%	5.36%	0.86
Race									
Not hispanic or latino	70%	71.7%	0.087	68.1%	68.7%	0.17	65.9%	67.4%	0.24
White	79.1%	80%	0.31	74.8%	75%	0.7	75.0%	76.8%	0.11
Black or African American	5.8%	5.92%	0.81	7.46%	7.46%	0.99	8.30%	7.27%	0.16
Hispanic or latino	5.18%	4.59%	0.22	4.87%	4.66%	0.3	4.41%	4.59%	0.74
Other race	2.39%	2.1%	0.37	2.61%	2.54%	0.63	2.61%	2.61%	1.0
Asian	1.01%	1.04%	0.91	1.48%	1.49%	0.97	1.03%	0.95%	0.78
American Indian or Alaska native	0.321%	0.247%	0.53	0.329%	0.352%	0.69	0.441%	0.44%	1.0
Native Hawaiian or other Pacific Islander	0.271%	0.247%	0.83	0.258%	0.231%	0.57	0.514%	0.70%	0.38
Unknown ethnicity	24.8%	23.7%	0.23	27%	26.6%	0.35	29.7%	28.0%	0.17
Unknown race	11.2%	10.6%	0.45	13%	12.9%	0.77	12.2%	11.2%	0.27
Comorbidities									
Essential hypertension	58.6%	59.2%	0.57	44.8%	34.3%	0.22	60.3%	61.4%	0.39
Hyperlipidemia	40.4%	39.9%	0.67	30.2%	21.8%	0.19	39.7%	39.1%	0.62
Overweight and obesity	27.7%	27.5%	0.8	23%	13.9%	0.24	35.6%	36.0%	0.78
Type 2 diabetes mellitus	17.9%	16.7%	0.14	17.3%	12.7%	0.13	24.9%	24.6%	0.85
Chronic ischemic heart disease	17.5%	16.3%	0.16	12.9%	8.62%	0.14	16.7%	15.7%	0.32
Hypothyroidism	10.2%	9.52%	0.3	8.41%	7.72%	0.026	14.8%	13.2%	0.086
Chronic kidney disease	7.3%	6.09%	0.029	4.68%	3.53%	0.058	10.3%	9.03%	0.12
Type 1 diabetes mellitus	2.81%	2.05%	0.025	2.04%	1.29%	0.059	4.15%	3.74%	0.44
Polycystic ovarian syndrome	0.617%	0.617%	1	0.703%	0.328%	0.052	0.881%	0.81%	0.77
Lab values									
Hemoglobin A1c	6.01 ± 1.44 42.2%	6.18 ± 1.56 41.2%	0.24	30.1 ± 6.6	30.8 ± 6.5	0.32	6.21 ± 1.65	6.27 ± 1.57	0.21
BMI	29.4 ± 6.18 74.8%	31.1 ± 6.39 76.5%	0.444	6.21 ± 1.59	6.21 ± 1.57	0.99	30.4 ± 6.93	31.7 ± 6.62	0.35

Among comorbidities, CKD was present in 7.3% of the alcohol cohort compared to 6.09% in controls (*P* = 0.029). Hypothyroidism was observed in 8.41% of the nicotine cohort vs 7.72% in controls (*P* = 0.026), and in the opioid cohort, 14.8% of cases compared to 13.2% in controls (*P* = 0.086). Type 1 diabetes mellitus was reported in 2.81% of the alcohol cohort vs 2.05% in controls (*P* = 0.025). Overweight and obesity were comparable in the alcohol cohort (27.7% vs 27.5%; *P* = 0.8), the nicotine cohort (23% vs 13.9%; *P* = 0.24), and the opioid cohort (35.6% vs 36.0%; *P* = 0.78).

### Outcomes

#### Alcohol

In patients with alcohol abuse or dependence undergoing laminectomy, several complications showed significant risks. At 90 days post-procedure, death (OR: 5.076, 95% CI: 2.727-9.448), DVT (OR: 2.041, 95% CI: 1.048-3.978), MI (OR: 2.283, 95% CI: 1.283-4.828), pneumonia (OR: 1.73, 95% CI: 1.026-2.917), sepsis (OR: 1.858, 95% CI: 1.157-2.984), and stroke (OR: 2.27, 95% CI: 1.074-4.8) were significantly elevated. At 1 year, acute renal failure (OR: 1.435, 95% CI: 1.064-1.938), death (OR: 5.114, 95% CI: 3.375-7.751), DVT (OR: 2.002, 95% CI: 1.209-3.315), pneumonia (OR: 1.755, 95% CI: 1.261-2.442), sepsis (OR: 1.821, 95% CI: 1.277-2.595), and stroke (OR: 1.878, 95% CI: 1.171-3.01) remained significantly elevated. Additionally, PE (OR: 1.932, 95% CI: 1.106-3.373) became significant at 1 year.

#### Nicotine

**Figure 3.**
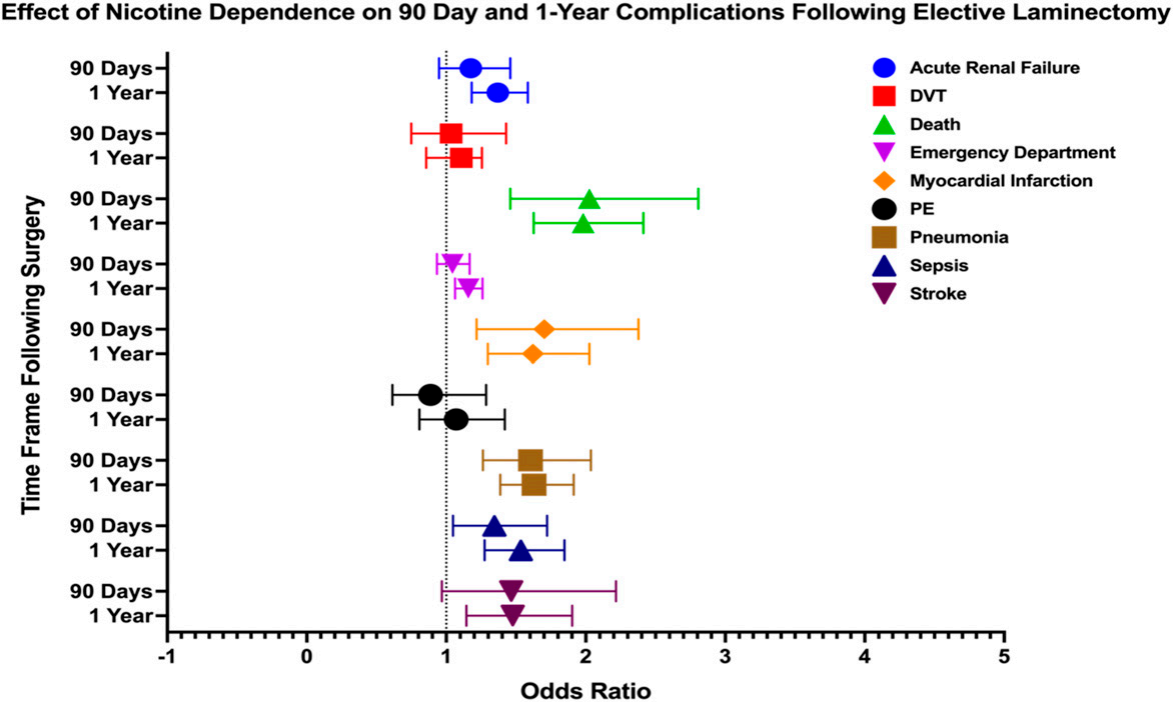
Forest Plot Depicting Impact of Nicotine Dependence on 90 Day and 1 Year Postoperative Complications Following Elective Laminectomy

In our analysis of nicotine’s impact on laminectomy outcomes, several complications were significantly associated with nicotine use at both 90 days and 1 year. At 90 days post-procedure, death (OR: 2.026, 95% CI: 1.458-2.807), MI (OR: 1.702, 95% CI: 1.218-2.378), pneumonia (OR: 1.603, 95% CI: 1.262-2.036), and sepsis (OR: 1.345, 95% CI: 1.049-1.723) demonstrated elevated odds. At 1 year, risks for several complications remained significantly high, including death (OR: 1.981, 95% CI: 1.626-2.413), MI (OR: 1.621, 95% CI: 1.298-2.026), pneumonia (OR: 1.629, 95% CI: 1.387-1.913), and sepsis (OR: 1.535, 95% CI: 1.275-1.847). Additional complications with sustained risks included acute renal failure (OR: 1.369, 95% CI: 1.182-1.585), stroke (OR: 1.476, 95% CI: 1.145-1.903), and ED visits (OR: 1.158, 95% CI: 1.064-1.26). Outcomes visualized via forest plot in Figure 3.

#### Opioid

**Figure 4.**
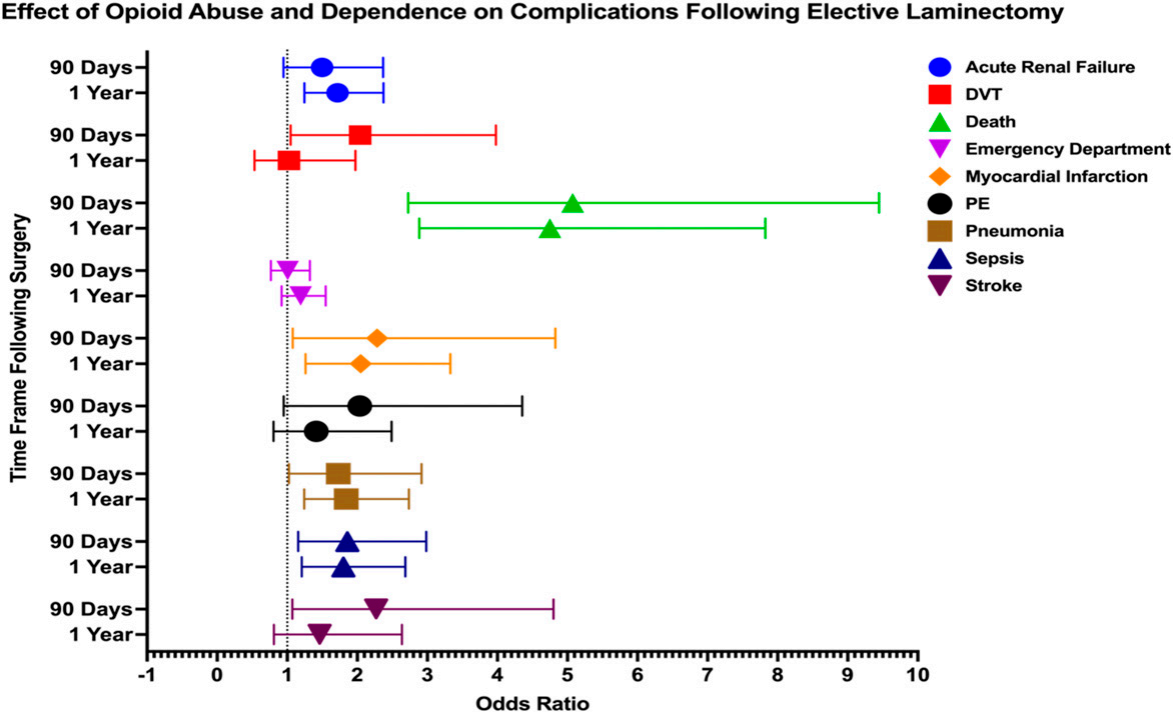
Forest Plot Depicting Impact of Opioid Abuse and Dependence on 90 Day and 1 Year Postoperative Complications Following Elective Laminectomy

In patients with opioid abuse or dependence, several postoperative complications showed significantly increased odds within both 90 days and 1 year following laminectomy. At 90 days, significant complications included DVT (OR: 2.041, 95% CI: 1.048-3.978), death (OR 5.076, 95% CI: 2.727-9.448), MI (OR 2.283, 95% CI: 1.080-4.828), pneumonia (OR 1.730, 95% CI: 1.026-2.917), sepsis (OR 1.858, 95% CI: 1.157-2.984), and stroke (OR 2.270, 95% CI: 1.073-4.800). At 1 year postoperatively, significant complications included acute renal failure (OR 1.719, 95% CI: 1.245-2.373), death (OR 4.751, 95% CI: 2.885-7.824), MI (OR 2.050, 95% CI: 1.262-3.328), pneumonia (OR 1.842, 95% CI: 1.242-2.736), and sepsis (OR 1.801, 95% CI: 1.208-2.686) (Figure 4).

## Discussion

### Comparison With Other Studies and Proposed Pathophysiology

#### Alcohol

In general surgical and orthopedic populations, high preoperative alcohol intake is associated with markedly elevated postoperative morbidity, including significantly higher rates of infections, impaired wound healing, and pulmonary complications.^5-9^ In spine surgery specifically, alcohol use disorders have been linked to increased perioperative complication rates and poorer recovery. For example, a nationwide study of over 3 million elective spine fusions found that patients with an alcohol use disorder – especially those experiencing acute withdrawal – had dramatically higher odds of major complications (respiratory, cardiac, thromboembolic, and wound-related) and nearly six-fold higher in-hospital mortality compared to non-drinkers.^10^ Additionally, Fineberg et al^11^ identified alcohol abuse as an independent predictor of postoperative ileus in a retrospective analysis of 220 522 patients undergoing various lumbar fusion approaches. In another study encompassing 578 457 lumbar decompression and fusion procedures, they also found alcohol abuse to be an independent predictor of postoperative delirium. Notably, however, not all studies have observed an effect with alcohol use. Elsamadicy et al^4^ reported no difference in 30-day complication or readmission rates between patients with and without a history of alcohol use. Shabanzadeh and Sørensen^12^ found no link to surgical site infections through their meta-analysis comparing outcomes of 440 drinkers vs 677 nondrinkers.

Our study contributes to this ongoing discussion by revealing that alcohol abuse or dependence significantly elevates the risks of complications following spinal surgery even without a fusion operation. Within 90 days, risks such as death, DVT, MI, pneumonia, sepsis, and stroke were significantly elevated, while at 1 year, the risks expanded to include acute renal failure and PE development.

Alcohol’s detrimental impact on surgical outcomes is well-documented, stemming from its extensive effects on healing processes and immune defenses. Both basic science and clinical research have shown that even acute alcohol exposure can dampen the injury response by reducing neutrophil recruitment and pro-inflammatory cytokine release, thereby delaying wound healing.^13,14^ Additionally, alcohol consumption can alter oral and sinopulmonary flora and suppress expectoration reflexes, thereby predisposing to infections like pneumonia and sepsis.^15-17^ With regards to acute renal failure, the significant liver dysfunction that arises secondary to chronic alcohol abuse hinders the metabolism of nephrotoxic substances, leading to acute renal injury. This insult can be exacerbated by concomitant hypotension, volume depletion, or rhabdomyolysis, which are common in chronic alcohol abusers.^18,19^ Thrombotic risks such as DVT, PE, and MI stem from the underlying liver-induced coagulopathy as well as the endothelial dysfunction that alcohol abusers can be at risk for. This risk is further heightened by prolonged immobility, which promotes venous stasis and creates a procoagulant environment conducive to clot formation.^20,21^

#### Nicotine

Nicotine smokers experience significantly higher rates of surgical site complications, including wound dehiscence, delayed healing, and infection, compared to nonsmokers.^22-25^ They also experience an elevated risk of impaired bone healing with the literature reporting that smokers have roughly double the incidence of pseudarthrosis after lumbar or cervical spinal fusions​ as compared to controls.^3,22-26^ Clinically, these factors translate into prolonged recovery and poorer patient-reported outcomes for smokers, who tend to report less improvement in pain and function after spine procedures.^27^ Moreover, smoking has been linked to a greater likelihood of additional surgery.^26^ One large series identified smoking status as the strongest independent predictor of reoperation following elective lumbar laminectomy, indicating that smokers are more prone to require revision procedures for recurrent degeneration. Beyond local healing issues, our findings confirm prior research reporting tobacco dependence predisposing patients to systemic complications.^28-30^

Cigarette smoking and nicotine exposure exert wide-ranging physiological effects that compromise surgical recovery through vascular, metabolic, and immunologic mechanisms. Nicotine induces persistent vasoconstriction, reducing peripheral perfusion and tissue oxygenation while also increasing myocardial oxygen demand, predisposing patients to perioperative ischemic events such as MI.^31,32^ Smoking also compromises pulmonary function and immune defenses, leading to impaired mucociliary clearance, reduced leukocyte activity, and heightened vulnerability to respiratory infections and sepsis.^33^ In the skeletal system, smoking impairs bone structure by increasing porosity and reducing density, thereby compromising overall bone integrity.^34^ Nicotine further disrupts bone metabolism by altering levels of cortisol and calcitonin and diminishing oxygen delivery, ultimately elevating the risk of fractures.^35-39^

#### Opioid

Preoperative opioid use has been consistently linked to worsened surgical outcomes in patients, including extended hospital stays, higher readmission rates, and increased healthcare costs.^40,41^ Mierke et al reported elevated readmission (34.2% vs 10.5%) and reoperation rates (34.2% vs 13.2%) in chronic users, while a broader review tied opioid use to heightened pain, postoperative complications, and even mortality.^7,42^ Our findings build on this body of evidence, highlighting the substantial risks associated with opioid use specifically in elective spinal laminectomy. At 90 days postoperatively, opioid users demonstrated significantly increased risks of complications, including DVT, death, MI, pneumonia, sepsis, and stroke. By 1 year, these risks persisted and extended to include acute renal failure, with mortality, MI, pneumonia, and sepsis remaining prominent complications.

Opioids heighten thromboembolism via coagulation changes and immobility, while immunosuppression by modulating opioid receptors in the nervous and immune systems increases infection risk.^43-48^ Nephrotoxicity from rhabdomyolysis and hypoperfusion drives renal failure, with chronic use linked to ongoing kidney issues, encompassing both acute and chronic kidney disease.^49^

#### Comparative Risk

Notably, our results demonstrated that patients with a history of alcohol use and those with opioid use exhibited similar odds of several postoperative complications, including mortality, MI, pneumonia, sepsis, stroke, and DVT, within 90 days after surgery. This similarity in outcomes likely arises from distinct, yet ultimately convergent, pathophysiological mechanisms by which these substances impair perioperative recovery. Alcohol use is well known to cause hepatic dysfunction, compromise immune defenses, disrupt coagulation, and delay wound healing, as shown in prior studies by Khan et al and Eliasen et al^5-50^ In contrast, opioid use is associated with immune suppression through mu-opioid receptor modulation, as well as respiratory depression, sedation, and decreased renal perfusion, as described by Luo et al^51^ Despite these differing mechanisms, both alcohol and opioid use lead to comparable vulnerabilities in organ systems, particularly affecting cardiopulmonary and infectious outcomes. Even when early postoperative complication rates appear similar, tailored approaches are warranted to address the unique ways in which alcohol and opioids compromise recovery.

Additionally, both alcohol use and opioid use were associated with higher postoperative complication rates compared to nicotine use at 90 days and 1 year. This aligns with prior literature demonstrating a broader systemic burden from alcohol and opioid exposure. Eliasen et al^5^ reported that high alcohol intake significantly increased the risk of infection, pulmonary complications, intensive care unit admission, and mortality, with relative risks up to 2.68 for death. Martin et al^52^ found that patients with opioid use disorder experienced higher rates of postoperative infection, readmission, and prolonged hospitalization. Nicotine use is associated with impaired wound healing, bone nonunion, and pulmonary complications, primarily through chronic endothelial dysfunction, microvascular vasoconstriction, and impaired fibroblast and osteoblast activity.^26-53^ However, it may not acutely disrupt systemic immunity or cardiopulmonary function to the extent observed with alcohol-induced hepatic inflammation or opioid-related respiratory depression. The resulting complications from nicotine tend to manifest more gradually and locally, rather than causing early systemic decompensation. These distinctions may explain the lower early complication rates seen in the nicotine cohort.

## Limitations

This study has several limitations due to its retrospective design and reliance on the TriNetX database. The use of ICD-10 codes for identifying diagnoses and complications may lead to misclassification errors, as these codes might not always accurately reflect patients’ clinical conditionsAdditionally, the TriNetX database may not capture all patient interactions, potentially resulting in incomplete data if individuals received care outside the network. Our analysis is further constrained by the lack of detailed clinical information beyond coded data, limiting our ability to assess factors such as the severity and duration of substance abuse, as well as withdrawal symptoms, which are important for understanding impact on surgical outcomes. Moreover, substance abuse data often rely on self-reports, which are susceptible to underreporting due to social desirability or recall biases. Unmeasured confounders, including socioeconomic status, medication use, and other social determinants of health, may also influence our findings, which may be a potentially large weakness of this study. Finally, as with all retrospective studies, the lack of randomization and prospective outcome tracking restricts our ability to draw causal inferences.

Despite these limitations, our study benefits from a large and diverse patient population, enhancing the generalizability of our findings. Future studies should aim for prospective designs that incorporate socioeconomic and clinical factors and enable more nuanced comparisons across multiple substance use groups for a deeper understanding of these impacts on surgical outcomes.

## Conclusion

In conclusion, our study highlights the substantial impact of substance abuse on 90 day and 1 year postoperative outcomes following elective laminectomy. Patients with histories of alcohol, nicotine, and opioid abuse or dependence exhibited significantly higher risks for complications such as MI, DVT, PE, pneumonia, sepsis, acute renal failure, stroke, and overall mortality. These findings underscore the importance of incorporating tailored preoperative risk assessments and perioperative care strategies for this vulnerable population. Future efforts should focus on integrating targeted interventions, including substance use cessation programs and enhanced monitoring, to reduce complications and improve surgical outcomes in patients with substance abuse histories.

## Data Availability

Data supporting the findings of this study are available from TriNetX but restrictions apply to the availability of these data, which were used under license for the current study and are not publicly available.*
